# Traumatic (?) Stricture of the Rectum*Reported to the Louisville Clinical Society.

**Published:** 1897-10

**Authors:** William C. Dugan

**Affiliations:** Louisville, Ky.; Professor of the Principles and Practice of Surgery and Surgical Pathology in the Louisville Medical College, etc.


### Southern Medical Record.

A MONTHLY JOURNAL OF MEDICINE AND SURGERY.

VOL. XXVII. ATLANTA, GA., OCT., 1897. No. 10

### Original Articles.

### TRAUMATIC (?) STRICTURE OF THE RECTUM.*

BY WILLIAM C. DUGAN, M.D., LOUISVILLE, KY.

Professor of the Principles and Practice of Surgery and Surgical Pathology in the Louisville Medical College, etc.

Mr. M., aged sixty years, was operated upon a year ago last March for an unusually bad rectal condition. Eighteen months ago I was asked by Dr. R. W. Taylor to see him for an abscess just below the umbilicus. The doctor had opened a number of them just above the bladder, and was rather suspicious of this one, fearing it might communicate with the bowel, and that in opening it a fecal fistula might result, and he wanted some one to see the patient with him, so that if a fecal fistula did result that he might not be censured for it.

At the time I saw Mr. M. I think he was the most miserable man I had ever seen. He had dwindled down to almost a skeleton, and we were afraid he could not stand the anesthetic should we decide to do an operation. He was passing his feces through a sieve-like opening, the result of an old traumatic (?) stricture; his bowels were operating almost continuously, in fact he was up night and day having stools. While we were considering the case he had us leave the room several times to allow his bowels to act. He would strain out a little hard fecal matter, which would relieve him for a few minutes, then it would be necessary for him to have another stool.

I suggested to Dr. Taylor that much could be done to relieve the patient by a colotomy. We suggested to Mr. M. the

* Reported to the Louisville Clinical Society.

advisability of that operation, and after a long consideration he consented to it. He was sent to the St. Joseph Infirmary and the operation was performed during March, 1896. He was very weak, as previously stated, at the time, and it was necessary to make the operative steps as short as possible. The usual incision was made, the bowel brought out and folded upon itself, and a lateral suture applied. The glass rod which is sometimes passed through the mesentery in performing this operation, was not found necessary, preferring the method practiced.

By an examination of the patient you will see here the opening, one leads upward, and the lower opening leading into the distal part of the sigmoid. It is advised by some operators to pass a double glass rod through the mesentery, to secure a good "spur," while others make a transverse incision severing the gut, then close up the lower opening. I did not do it in this case, and do not believe it is advisable. I do not know what Dr. Matthews will say about this feature, but I am satisfied it is better to leave it as it is, because we may have another abscess below as a result, and it certainly gives him no inconvenience as it is. He wears a pad over the opening as you see. You cannot appreciate the difference in the man's general appearance now and before he was operated upon. He says he is having four stools a day. If he would take a little pains with himself and use an enema into the upper opening, he would have a stool in the morning, or perhaps two a day, but he has not taken that precaution and simply lets the actions come as they will. He is the most sensitive, modest man that I ever met, and it was only because he felt under obligations to me that he came before us tonight. He says he has gained twenty-five pounds in weight since the operation. To a man of his build that means a great deal. He has had a great number of abscesses below the colotomy opening, and you will observe the remains of numerous fistulous tracts through the scrotum, buttocks, perineum, etc.

For some time after the operation he suffered from severe melancholia; he would wake up at night frequently and remark to his wife that "he had that opening in his side." This was one of the most distressing features of the case. He often said to his wife that he would rather be dead, and it was a long time before he could reconcile himself to this "nuisance," but the first time I saw him afterward he came to my office with his wife, and told me he had gotten over his nervous melancholic condition. He now suffers no mental disturbance.

My object in asking Mr. M. to come here was to bring up the subject of colotomy in these desperate cases of multiple fistulæ and rectal strictures. I am satisfied there are a great many patients who are carrying traumatic or syphilitic strictures of the rectum, who are a misery and burden to themselves, and who could be made much more comfortable by this operation of colotomy.

You will notice a great deal of bulging of the gut, and I wish to say that is the condition desired, and this is the difference between an artificial anus and a fecal fistula. In fecal fistula where you have it discharging through a small opening, there is a constant oozing of fecal matter, in striking contrast to those where you have simply an oozing of mucus. This man has regular fecal action through the wound, and a discharge of more or less white of egg looking substance—mucus—all the time.

This bulging is the lining of the gut and stands as a guard against any possible passage of fecal matter from the proximal to the distal opening, as this mass is between the two openings, in fact is the so-called "spur."

The history is that he was struck on the anus twenty-five years ago by a companion, and dates his trouble from that blow. He says that he usually had, during these twenty-five years, twenty to forty actions of the bowels every day. He has never had any stricture or other form of bladder trouble, except some little difficulty in passing his urine at different times. Since the operation of colotomy he has had no symptoms referable to the bladder.

Of course there is nothing new in the operation performed upon this man, and he is here but to show what can be accomplished by this unpopular operation; and I wish to say again that it is a much neglected operation and that there are a great many patients on the streets to-day that ought to be so operated upon. Their lives would be made more comfortable—in fact, restored to usefulness, and I may say, to society, as in the case of Mr. M.

As the dressing and pad were removed, you noticed no odor of fecal matter about this patient; in fact, there is no oozing of fecal matter whatsoever—simply a discharge of mucus from the colotomy opening. He has some discharge of mucus, also, from the rectum, making it necessary for him to wear a dressing. There is no discharge of pus from either the colotomy wound or the rectum. I do not know what can be done to improve this condition, whether irrigation from above down through the rectum with a saline solution would do any good or not, but as he is doing so well, I think it advisable to simply let him alone.

He had rather a stormy convalescence, as stated, from the fact that he deepened into a melancholia and wanted to die; he gave us but little assistance, and it was only by the persistent care of his wife and the patient's vitality that carried him through. As stated, after he went home from the Infirmary the melancholia continued for some time, and we were apprehensive as to his mental condition, but he finally regained his physical condition, and, at the same time, his mental equilibrium.

Whether the original trouble was traumatic or specific, I am unable to say, as the previous history of the case is rather indefinite and unsatisfactory. However, he absolutely denies any history of specific disease. He states that he was sitting over a chair, with his face to the back of the chair, and was struck over the anal region. The blow was not followed by an abscess, but he suffered great pain at the time, which persisted for several months before he solicited medical aid.

DISCUSSION.

DR. W. O. ROBERTS: I agree with Dr. Dugan that the operation of colotomy is not done as often as it should be, and the result in the case before us shows the wisdom of it. It also shows the advantage of performing the operation in the inguinal rather than the lumbar region. The patient can handle the colotomy opening without any trouble; he keeps it perfectly clean. I believe, however, that closure of the lower portion of the gut should always be done. If an abscess should develop afterward, as stated by Dr. Dugan, drainage below could be resorted to.

DR. J. M. MATHEWS: About fourteen years ago this gentleman called at my office, and even at that stage of the disease I confess that I had never seen a case equal to it. He had fistulæ extending out into the buttocks, into the perineum and into the scrotum; there were a great many of these fistulous tracts. I did not count them, but there must have been thirty or forty. I introduced my finger into the rectum and found an obstruction, which, if Dr. Dugan will permit me, I do not believe was traumatic. The strictures then extended as far as I could reach. A traumatic stricture usually, when from a blow especially, originates in an abscess, cicitrization takes place, and as it proceeds, narrows the lumen of the gut, only forming a cicatrix or stricture at the place where the blow was received. That character of stricture is easily overcome by simply dividing it; it is a fibrous band. I tried to trace the origin to syphilis, but he gave no specific history, though he would not positively deny it. He was at that time a railroad man, and probably had ample chances for infection, and we know from the history of syphilis that many cases are brought to the physician who deny having had it, but really they did have it. A very small chancre may occur without attracting attention of the patient, except the knowledge, perhaps, that he has had a small sore on his penis. I believed then that this man suffered at that time from syphilis of the rectum. It certainly is not a simple stricture. The whole of the rectum is involved clear to the sigmoid flexure, and was fourteen years ago. The fecal mass could not pass, and therefore he had an ulcerative process first, then the fistulæ developed. I said to him at the time that he should have a colotomy performed.

As to the point made by Dr. Dugan and indorsed by Dr. Roberts, I am on record as being rather opposed to colotomy. I mean by that and desire to say that I believe if not in this section, in a great many other sections of the country, too many colotomies are performed, rather than too few. In explanation of this I want to say: I have made the point repeatedly that a typical case for a colotomy is a case like this. Here is a man that gives no evidence of malignancy; it amounts to simply an obstruction in his rectum; that if you will open the colon and allow this man to pass his feces in that way, he will live just as long as he would if he had no obstruction. I will indorse what the previous speakers have said if they will make it read that a colotomy should be done for total obstruction of the rectum from a syphilitic stricture, and want to say that there are more syphilitic strictures of the rectum than we imagine. There are many patients whose rectums are blocked up by a syphilitic deposit and who will die of sub-acute peritonitis or something of that kind, where the condition is often entirely overlooked by the physician or surgeon. I see them often, and upon these cases a colotomy should be performed. It is sometimes an obstruction above the reach of the finger, and in such cases especially where the trouble is *syphilitic*, a colotomy should be done.

I am glad Dr. Dugan has presented this case for several reasons. I am satisfied if the patient had been presented to some men, in some sections of the country, that it would have been pronounced at the time I saw it a malignant growth. This man was emaciated, he had practically no flesh on his body, he was in a miserable condition, he even had sweats, had no appetite, etc. I told him he would not live a year, and am surprised that he has lived so long. I was honest in the statement; I did not believe the man would live a year. At that time he used the expression as quoted by Dr. Dugan tonight, that he would rather die than have a colotomy performed upon him. Dr. Senn, of Chicago, whom we regard as one of the most eminent surgeons in this country, said at one time that he would not perform a colotomy, that it was the most disgusting operation he ever heard of, or had ever witnessed, and he believed that it did very little good. Since then Senn has performed some colotomies but not a great many. In the East such men as Bull and McBurney told me they very seldom performed colotomy, but I believe they referred to the class of cases to which I have called attention, viz: cancerous patients. I condemn colotomy in the vast majority of cancerous patients for reasons which I have frequently given.

Dr. Dugan evidently recognizes that there is too much bulging at the colotomy wound in this case. Now could it have been prevented? I think it could. I believe in running a glass rod through the mesentery; I have recommended the running of two glass rods through the mesentery for the sole purpose of securing a good spur. This is absolutely requisite, and Herbert Allingham brought himself into notoriety by opposing the operations done by other men for their lack of making this spur. A German authority quoted by Senn in his book proposes a new method of doing a colotomy which I have practiced once. That is to bring the gut out and instead of running a rod through underneath, or adopting the Cripps operation of attaching the mucous membrane to the true skin, pulling out several inches of the gut and bringing it together and sewing it there so as to hold the gut up very high, then opening the gut, so that you do not have the discharge of any feces below. One remarkable thing to me has always been in doing a colotomy is that when I first began doing the operation I had the idea that it cut the feces entirely off. You know that is the point that has been fought by Allingham about his method, that you do not have as much fecal matter passing down into the rectum. But the vast majority of patients upon whom a colotomy has been performed say they still have repeated actions from the rectum, and in most cases feces will pass down over the colotomy into the rectum. Either by means of the glass rod or by pulling out a greater amount of the gut and stitching it as described by the German authority referred to, this feature may be largely overcome.

In closing I must say that this case does demonstrate what Drs. Dugan and Roberts have stated, that the operation of colotomy is not performed often enough in this class of patients, where it is traumatic or syphilitic. Every single one of them in my opinion should be colotomized for the simple reason that no syphilitic rectum ever got well after the formation of fibrous strictures. I do not believe there is one on record; you can break, or cut, or curette them, but you never cure the patient. Therefore I say every one of them should be colotomized if they will submit to the operation. It is a question with some intellegent people whether they will submit to a colotomy; I have often met people who would not consent to the operation. But from a surgical standpoint I believe that these two surgeons, when they state that the operation is not done often enough in this class of cases, will agree that it is performed too frequently for malignancy or for simple stricture of the rectum. Certainly in malignancy the life of the patient is not materially prolonged, nor can it be considered a proper operation.

DR. WM. CHEATHAM: I believe I would rather be dead than to be in the condition presented by the patient who has just been before us. I want to ask Dr. Mathews whether an intubation tube could not be used in this class of cases where there is a stricture of the rectum. We often see syphilitic stricture of the larynx and upper part of the respiratory tract for which intubation is practiced; why may it not be used in rectal stricture? An intubation tube of proper size and shape could be introduced through the stricture and could be worn continuously. I would like to ask if this method has ever been practiced?

DR. J. M. MATHEWS: An objection to the method suggested by Dr. Cheatham would be the almost continuous drainage of fecal matter that would necessarily take place. If a tube could be arranged with a valve, and could be held in position by some means, I think the suggestion would be feasible.

DR. W. C. DUGAN: In performing the operation upon this patient, the bowel was brought up to make a spur as Dr. Mathews has suggested. In regard to prolapse, the way to prevent this is by drawing upon the proximal end of the gut, bringing it down as far as you can before applying the sutures. That is the method that was followed in this case. When the bowel was brought out, the method suggested by Dr. Mathews of putting in two sutures on each side through the serous coats was practiced. Then the gut was sutured all the way around. I preferred that method to the use of the pins or rods as recommended by many operators. The gut was brought out so as to make a good spur, two sutures were introduced in each side holding the gut, then the gut was sutured all the way around and left out. I left it for two or three days, then took off the dressing, and without the administration of chloroform or local anesthetic made a transverse incision. A transverse incision was made because I knew it was going to be permanent, instead of making a longitudinal section as might be done when it is the intention to return the bowel to the cavity, at some future time.

In regard to the pathology: I am inclined to regard it as syphilitic, as I do not see how such an extensive stricture could occur otherwise. I could make no examination of the interior of the rectum when I saw him, for the reason that the rectum was completely closed; it was even impossible to introduce an ordinary sized probe. There were numerous fistulous openings, the remains of which you saw tonight, and it was through these he had his stools. He had on his abdomen two fecal fistulae that were discharging pus and decomposed fecal matter all the time, and he smelled like a badly kept privy—this was the principal reason for submitting to the operation. After considering the case carefully, I advised the operation and he reluctantly submitted. I am satisfied, as he stated tonight, that he would not have submitted to the operation, had not the disgusting condition existed.

Although there is no history of syphilis in the case and his wife and several children are free from it, yet I feel satisfied that the condition is specific in origin. The history of trauma that I have given comes from the patient himself, and I am unable to say just how much of it explains the pathology.

